# Archetypes of Footfall Context: Quantifying Temporal Variations in Retail Footfall in relation to Micro-Location Characteristics

**DOI:** 10.1007/s12061-021-09396-1

**Published:** 2021-07-28

**Authors:** Susie Philp, Les Dolega, Alex Singleton, Mark Green

**Affiliations:** Department of Geography & Planning, University of Liverpool, Liverpool, U.K.

**Keywords:** Retail, Footfall, Town centre micro-locations, Cluster analysis

## Abstract

The UK retail sector is constantly changing and evolving. The increasing share of online sales and the development of out-of-town retail provision, in conjunction with the 2008–09 economic crisis, have disproportionately impacted high streets and physical retail negatively. Understanding and adapting to these changes is fundamental to the vitality, sustainability and prosperity of businesses, communities and the economy. However, there is a need for better information to support attempts to revitalise UK high streets and retail centres, and advances in sensor technology have made this possible. Footfall provides a commonly used heuristic of retail centre vitality and can be increasingly estimated in automated ways through sensing technology. However, footfall counts are influenced by a range of externalities such as aspects of retail centre function, morphology, connectivity and attractiveness. The key contribution of this paper is to demonstrate how footfall patterns are expressed within the varying context of different retail centre architypes providing both a useful tool for benchmarking and planning; but also making a theoretical contribution to the understanding of retail mobilities. This paper integrates a range of contextual data to develop a classification of footfall sensor locations; producing three representations of sensor micro-locations across Great Britain: *chain and comparison retail micro-locations, business and independent micro-locations* and *value-orientated convenience retail micro-locations*. These three groups display distinct daily and weekly footfall magnitudes and distributions, which are attributed to micro-locational differences in their morphology, connectivity and function.

## Introduction

The retail landscape in the UK is constantly evolving. In 2019, 19.2% of retail sales were made online; an increase of 13% over 10 years (ONS, 2020). The increasing share of online sales, in conjunction with the 2008–09 economic-crisis, the development of out-of-town shopping retail space provision and shifting consumer behaviour, are major drivers for retail industry change, and physical retail has suffered disproportionately as a result (Burt, 2010; Parker et al., 2016a; Portas, 2011; Wrigley et al., 2015). This period of retail upheaval has had significant consequences, especially for those businesses who have failed to adapt to changed consumer purchasing behaviour and online competition. Recent examples include Clintons and Forever 21 who, along with 41 other retail chains, went into administration in 2019 (Centre for Retail Research, 2020). With what were once household names disappearing from the high street, concern has cultivated within media, public opinion and government on what this means for the future of the retail industry and the UK economy.

There is a consensus that data driven empirical evidence is needed to support high street performance and revitalisation strategies (Portas, 2011; Wrigley & Dolega, 2011). In particular, footfall, often cited as the '**lifeblood**' of a high street vitality and viability (Birkin et al., 2017), is a key measure for the successfulness of these strategies and a widely used proxy for their economic performance (Coca-Stefaniak, 2013; Millington et al., 2018). Footfall can be defined as the count of people travelling through a shopping area at a given point in time (Lugomer et al., 2017). As a measure, footfall is responsive to both characteristics of the macro-scale environment, such as broader economic trends, catchment population or weather conditions (Dolega et al., 2016; Makkar, 2020), and the micro-scale environment, referred to as the micro-location. Micro-location analysis recognises the influence of the immediate environment on footfall, for example, the mix of retailers along a street or walkability (Brown, 1993) as such, larger retail centres can encompass multiple micro-locations. There is limited research detailing or quantifying the relationships between footfall and qualities of the micro-location, resulting in low understanding of the opportunities and pitfalls footfall data may present. This can have implications for decision makers, who may use footfall as a primary measure of high street vitality and viability, and for the understanding of retail mobilities as a whole. As such this paper uses quantitative data to investigate the relationship between patterns in footfall and the function, morphology and connectivity of retail micro-locations by fulfilling three key objectives: i) create a classification of the micro-locations based on the functional and morphological properties; ii) identify the key characteristics of these micro-location clusters and iii) examine how the temporal footfall patterns vary across different micro-location clusters.

This paper continues as follow. In Sect. 2, the importance of footfall as an indicator for retail centre vitality is discussed in addition to identifying retail centre qualities which determine footfall. Section 3 concerns the data collection, derivation and analytical approach used to cluster the 640 micro-locations across Great Britain into three representative clusters. These clusters are investigated in terms of their different attributes and their average footfall distributions in Sect. 4 and in Sects. 5 and 6 the different processes behind these results and their implications are discussed.

### Retail Centre Vitality and Footfall

Retail centre vitality is a term used to reflect the liveliness of a retail centre and is measured by its busyness both across space and time (Parker et al., 2016b). There has been a wide range of normative studies into retail centre vitality, though, as a result of the negative impact of recent changes in the retail sector, there has been an emergence of more critical research (Parker et al., 2016a). Efforts by the government and private sector have aimed to understand the challenges which high streets are facing, and how they can adapt to succeed in the future (Coca-Stefaniak, 2013; Grimsey, 2018; Parker et al., 2016b; Portas, 2011). There is a general consensus that sustainability and prosperity can be found through cooperation of stakeholders towards a clear and accountable vision (Coca-Stefaniak, 2013; Grimsey, 2018; Portas, 2011). However, evidence suggests that there are limited examples of successful application of these practices (Parker et al., 2016a; Wrigley et al., 2015). To establish sustainable retail environments for the future, it is key to understand what impacts vitality (Coca-Stefaniak, 2013; Parker et al., 2016a). Retail centres can be viewed as complex economic systems, and as such their vitality is driven by a number of internal and external factors, such as attractiveness, diversity and accessibility (Parker et al., 2016b).

There is also a plethora of research that investigates various measures of retail centre economic performance. A common measure is vacancy rate (Wrigley et al., 2015) and its derivatives such as vacancy rate change, structural vacancy and spatial clustering of vacant units. Retail offer and commercial rents are also commonly used for finer-scale performance insights (Wrigley et al., 2015). Another commonly used heuristic in academia, industry and in government for vitality and sustainability of a retail centre is footfall (Coca-Stefaniak, 2013; Millington et al., 2018). Footfall was identified as the most influential factor for high street vitality and viability by Parker et al. (2016a) as a result of consulting 22 retail experts for their insights. Research suggests that this could be in part due to the positive correlation between footfall and potential spend (Graham, 2017; Koster et al., 2019; Warnaby & Yip, 2005), which in turn, can be linked to high return on investment for stakeholders, consequently attracting future investment and creating economically viable retail centres (Graham et al., 2019).

Footfall is also a proxy for the vitality of a retail centre beyond consumer spend. It can be used to capture the attractiveness of a location as a community hub, workplace or other social and communal functions which a retail centre can provide to its consumers (Millington et al., 2015). A clear example of this is Edinburgh, a city ranked 3^rd^ in the UK for footfall, however only 12^th^ in terms of actual spend (Millington et al., 2015). This shows that there is a proportion of Edinburgh’s footfall that does not translate into spend. The utility of footfall as a measure that encompasses many different influences and processes of the retail environment makes it a beneficial and useful indicator of retail vitality and viability.

#### Determinants of Footfall

**Fig. 1:**
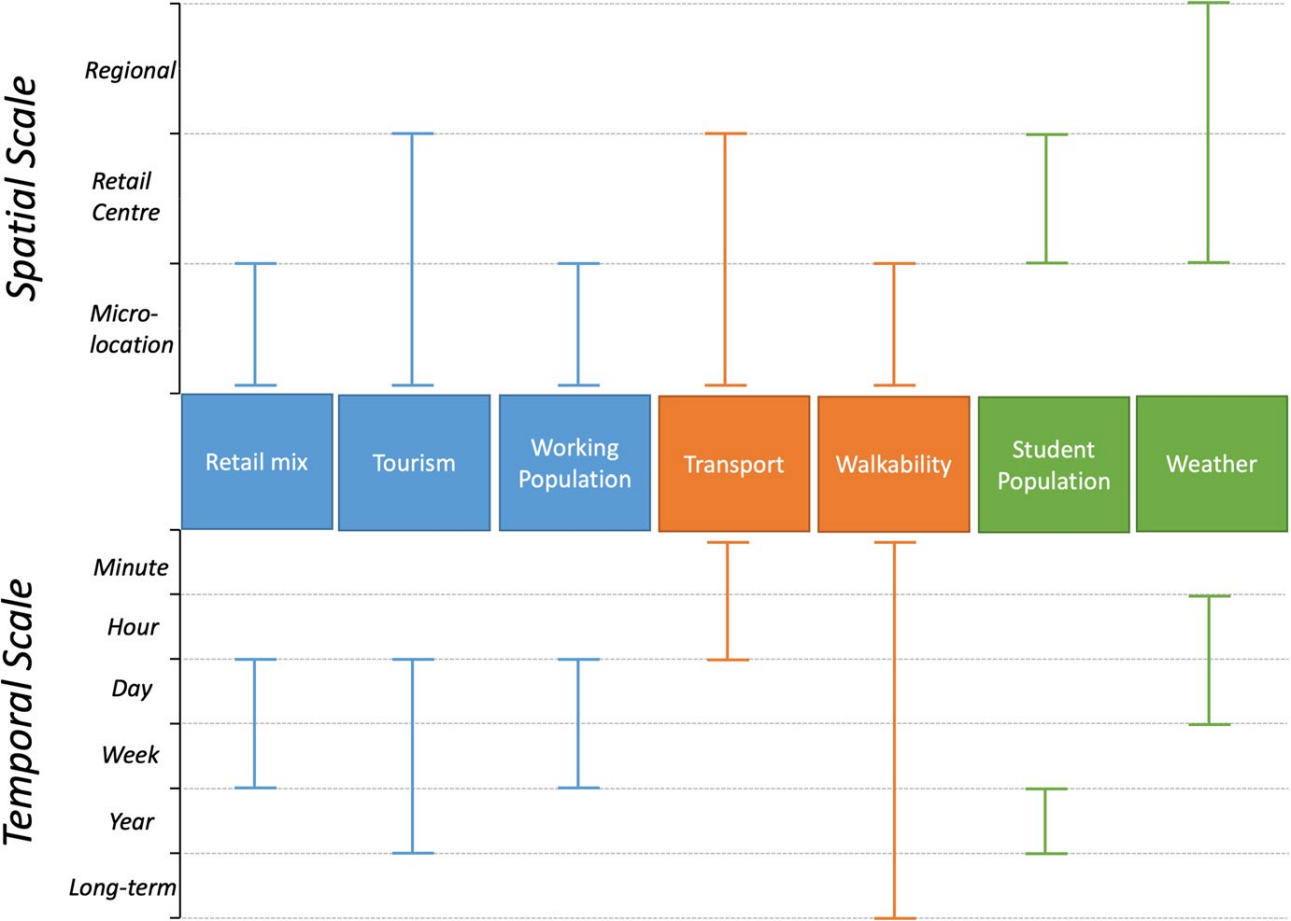
Diagram summarising the spatial and temporal impacts of different footfall determinants as discussed in Sect. 2.1

Footfall is determined by a multitude of factors on different spatial and temporal scales, visualised in Fig. 1. Here, these determinants are summarised under three main headings: functional, morphological and other. The factors which influence footfall are interrelated, complex and can be difficult to quantify. This comprehensiveness can present a problem when trying to explain temporal and spatial variations in magnitude and signature. The magnitude of footfall can be defined as the amount of people measured in a certain set time period and the signature refers to the variation of footfall magnitude over time.

#### Function

As shown in Fig. 1, footfall is influenced by a multitude of factors on different temporal and spatial scales. Working population, retail mix and tourism all influence micro-location footfall to a daily, weekly or seasonal temporal scale and relate to the retail centre function. The function of a retail centre is the purpose which it serves to users and most retail centres are multi-functional, simultaneously performing several purposes (Millington et al., 2015). Characteristics such as the presence of anchor stores or the tendency towards premium or value goods can all indicate the retail centre identity, who it may appeal to, and consequently, when they may visit (Guy, 1998).

The function of a retail centre or micro-location impacts footfall in several ways. Firstly, having a varied and cohesive retail mix has been shown to boost retail centre vitality and attractiveness (Millington et al., 2015; Tyler et al., 2012). The better the ability of the micro-location retail offer to match consumer demand of the consumers, the busier it can become, increasing the magnitude of footfall (Parker et al., 2016a; Portas, 2011).

Secondly, research shows that the function of a retail centre is closely aligned to both diurnal and other periodic patterns of use. For example, retail centres in locations with a high concentration of employers and businesses typically have higher daytime footfall (Berry et al., 2016; Swinney & Sivaev, 2013). Such relationships have been shown to drive footfall and sales during weekdays, especially in the early morning, at midday and in the early evening (Berry et al., 2016). On a seasonal scale, tourist destinations such as Cornwall can see grocery retail demand double during on-season (Newing et al., 2018) with tourists that are likely to spend more than local customers (Newing et al., 2014). In addition, event-based tourism can drive footfall on a more short-term basis. For example, the Giant Spectacular Liverpool’s Dream event drew in 1.3 million people over 4 days in October 2018 (giantspectacular.com, 2019).

Thirdly, studies which have investigated temporal change in footfall signature and magnitude have explained their results by primary retail centre function. Mumford et al. (2017) identified four distinctive annual footfall distributions for the UK, attributing their differences to four functions: comparison retail, holiday destinations, speciality retail and a multifunctional purpose. Similarly, in Lugomer and Longley (2018), footfall data was clustered based on the hour of the day, resulting in nine different patterns which were partly explained by different primary functions.

#### Walkability and Morphology

Another factor which influences footfall is walkability, impacting micro-locational footfall over multiple temporal scales (see Fig. 1). There are many contesting definitions of walkability however, in this case, walkability can be defined as the attractiveness of a street to a pedestrian. This can pertain to physical characteristics, security, network connectivity and transport connectivity (Lo, 2009). Indeed, certain morphological properties of streets have been shown to increase their walkability, such as wide streets with gentle slopes that are well lit have been shown to be the most attractive (Erath et al., 2017; Unwin et al., 2017).

Additionally, how the street is situated within the wider network has proven to be a reliable indicator of pedestrian counts (Hillier et al., 1993; Raford & Ragland, 2006). In particular, well-connected streets tend to have higher footfall as it is often the shortest route from their origin to their destination. This can be determined by various measures of centrality including closeness and betweenness, which respectfully capture the closeness of a node to other nodes and the prominence of a node as a bridge between other nodes (Freeman, 1977; Porta et al., 2009). As such, they can be used to predict busy intersections, or nodes. The added benefit of betweenness centrality, as opposed to closeness centrality, when investigating pedestrian flows is that it can be calculated for the edges, or streets, as well as the nodes.

Streets can also have high walkability if they are close to access points for other forms of transport, such as train stations, car parks or bus stops (Mazumdar et al., 2019). As popular origins and destinations, these features can concentrate footfall to particular micro-locations (Scheurer & Porta, 2006). Anchor stores, restaurants and entertainment venues have demonstrated footfall attraction in a similar fashion (Hart et al., 2014; Koster et al., 2019; Teller & Alexander, 2014; Üsküplü et al., 2020; Yuo et al., 2003). The proximity of stores to major transport hubs has been shown to increase their footfall and sales, particularly at commuting times (Berry et al., 2016). Having good access to car parking is a demand of retail areas and many consumers will avoid using public transport in favour of the convenience of their own vehicle. Therefore, the proximity of a retail area to a public car park can influence the quantity of visitors and impact footfall for the entire retail centre (ATCM, 2014).

#### Additional Factors

In addition to walkability and function, there are numerous other factors which have been proposed to influence the magnitude and distribution of retail centre footfall, for example weather, with rain and snow drastically reducing daily pedestrian counts (Makkar, 2020). Although extreme weather is typically a dynamic and short-term influence, it can have significant consequences, particularly if it coincides with planned periods of high expected retail, such as the Christmas season.

Academic literature points to many functional and morphological influences on footfall, however, to our knowledge, no literature exists which quantifies the impact of a combination of these influences. Therefore, a data driven exploration of footfall spatial and temporal patterns will add quantifiable evidence to the existing evidence base in this research area, in particular to observed relationships between footfall and the characteristics of the surrounding micro-location.

### Methodology

#### Footfall Data

Footfall data were provided by the ESRC Consumer Data Research Centre / Local Data Company [LDC] whose sensors use probes from Wi-Fi enabled devices to estimate the number of smart phone devices passing by as a proxy for footfall. The device sends an individual MAC address to the sensor, which is anonymized and used to determine which kind of device the signal came from. Devices which are not smart phones are filtered out, as are duplicate counts from residents or staff nearby by filtering out MAC addresses that appear in several chronological time periods. The counts are aggregated to 5-min intervals.

The approach relies on some assumptions which may limit its accuracy (Lugomer et al., 2017; Soundararaj et al., 2020). Firstly, a count of smart phones is not a perfect count of people as not everyone owns one or has one with them as they travel around a retail centre. Secondly, as the battery of a phone gets lower, it does not send out the wi-fi probes as far or as often, making it less likely to be picked up by the sensor than if it was on full battery. Thirdly, if a pedestrian has their Wi-Fi switched off, depending on the model of the device, the sensor may not register them. Fourthly, due to increased phone security implemented within newer phone operating systems, MAC addresses are scrambled a lot more frequently, making it harder to filter out repeat counts. Furthermore, there can be practical issues which cause measurement inaccuracies such as power cuts, sensors being mistakenly switched off or differences in positioning and orientation of the sensor.

A number of measures have been taken to overcome these problems. Before any analysis was run on the footfall counts the measurements were compared to manual counts. These manual counts take place for every location at a range of times throughout the day, month and year to ensure that the footfall counts are adjusted as reliably as possible (Soundararaj et al., 2020).

As of August 2018, LDC had sensors in 840 locations in 88 towns and cities across the UK (LDC, 2018). Due to data availability restraints, the study used 640 sensors from 40 high street retail locations in Great Britain. The distribution of sensors is particularly biased towards London (*n* = 291), with 45% of the sensors, as well as larger cities such as Manchester (*n* = 18), Liverpool (*n* = 16) and Cardiff (*n* = 8). Excluding London, the number of sensors per location ranges from *n* = 20 in Kingston-upon-Thames to *n* = 1 in Gateshead and in Windsor. Although the majority of sensors in the sample are in larger cities, some regional centres and market towns are also represented, such as Taunton (*n* = 6) and Market Harborough (*n* = 13). The full geographical distribution of the sample can be found in Appendix A.

#### Derivation of Footfall Descriptors

Drawing on previous work identified from the literature review, we can draw a series of broad micro-locational influences on footfall that are related to: ‘functionality’ and ‘morphology and connectivity’. Within each category, there are a range of potential variables that can be assembled to differentiate between the footfall sensor micro-locations. By understanding the differences in footfall descriptors between the footfall sensor locations, elements of their footfall magnitude and signature can be better inferred. The descriptors used are not an exhaustive list of footfall influencers, therefore this analysis relies on the assumption that the impact of other influencers is negatable.

A summary of the variables within their category and their specification are shown in Table 1.

**Table 1:** Key features of the functionality and morphology and connectivity variables used as micro-location footfall descriptors

Category	Variable	Specification
Functionality	Distance to the nearest anchor store	Euclidean distance (metres) to nearest anchor store, identified by their brand name (e.g. John Lewis, Primark, Debenhams, full list in Appendix C)
	Distance to the nearest premium store	Euclidean distance (metres) to the nearest premium store, identified by their brand names (e.g. The White Company, Burberry, full list in Appendix C)
	Distance to the nearest entertainment activity	Euclidean distance (metres) to the nearest venue which offers an entertainment activity (e.g. Cinemas, Arcades, Museums). These were identified using the LDC (2017) survey sub-categorisation (full specification in Appendix C)
	Proportion of vacant stores (vacancy rate)	The proportion of vacant store identified using the LDC (2017) survey within a 100 m straight line buffer of the sensor
	Proportion of value stores	The proportion of stores identified as value stores by their brand name (e.g. Aldi, Home Bargains, full list in Appendix C) within a 100 m straight line buffer of the sensor
	Proportion of independent stores	The proportion of stores identified as independent by the singular instance of their store name in the dataset within a 100 m straight line buffer of the sensor
	Proportion of night-time economy locations	The proportion of locations within a 100 m straight line buffer of the sensors which offer a typical evening appeal (e.g. bars, clubs, restaurants, fast food) identified using LDC (2017) survey categorisation (full specification in Appendix C)
	Workplace population	The average of the daytime population densities of the workplace zone in which the sensor falls into, and those which border it (ONS, 2017)
	Ratio of service to retail	The ratio of the locations within a 100 m straight line buffer of the sensor which are identified as service locations by LDC (2017) survey classifications to those identified as comparison retail and food retail (e.g. grocery stores, butchers, confectioners, further specifics in Appendix A)
Morphology and Connectivity	Distance to the nearest transport hub	Euclidean distance (metres) to the nearest group of bus stops or train station as identified in the NaPTAN dataset (Department for Transport, 2014)
	Distance to the nearest car park	Euclidean distance (metres) to the nearest car park as identified by the Department for Transport (2015)
	Density of stores	The number of store units within a 100 m straight line buffer of the sensor
	Centrality of the street	The street centrality measure was calculated from networks generated by the OSMnx python library. OSMnx uses data from Open Street Map to generate a network graph of a road structure within a boundary. The CDRC retail centre boundaries (Pavlis et al., 2017) were used to generate the pedestrian network around a sensor. The edge betweenness centrality of the street which the sensor was on was is then calculated to give the street centrality measure. Edge betweenness was chosen as the centrality measure because it can be applied to streets instead of intersections, where most of the footfall measurements are taken from. This captures the prominence of a street as a pass-through route

The Functionality category captures aspects of context that may attract people to a retail area. The purpose for patronage of a retail area is logically linked to a temporal factor, for example, food outlets will attract more people during mealtimes and an area rich with bars and restaurants, would attract people in the evenings aligned to opening hours.

The morphology and connectivity category encompasses features of walkability and attractiveness such as transport accessibility, density of units and the centrality of the street within the retail centre network.

For several descriptors, a 100 m circular buffer^1^ around the sensor was used to select the stores close enough to be considered within the immediate retail environment of the sensor. 100 m was chosen as it encompasses a reasonable sample of stores to derive a full picture of the retail environment but is not so large as to remove the micro-locational variation of interest. This relies on the assumption that there is a dense concentration of retail units around the store the sensor is based in, and that the circular shape can appropriately capture this. Sensors with fewer than 5 units within the buffer area (total of 5 sensors) were removed from the sample as there are not enough stores to get a representative understanding of the proportions within the retail environment. The resulting number of stores in the buffer ranged from 7 to 189, which was used to define the density of stores variable. This was combined with the number of features such as independent and value stores to calculate proportions to represent these characteristics. Also, a proportion of vacant units was calculated within each buffer to obtain vacancy rate for each micro-location.

A Euclidean distance, as opposed to a proportion, was calculated for some features, such as anchor stores and premium stores, as they appear in most retail centres, though not in multitude. When a proportion was calculated for these features, they returned measures with more constrained variation. As such, distance was deemed to be a more appropriate measure. Table 1 below provides a summary of the variables, their specification. The correlation coefficients between these variables are shown in Appendix B.

#### Analytical Approach

Understanding how the footfall descriptors derived in Sect. 3.2 relate to the footfall magnitude and signature for their sensors is a complex and multi-dimensional task. For each of the 640 sensors, there are 13 functional and morphological descriptors which could impact their footfall magnitude and signature at different times of day and days of week. Although this density of data would be beneficial for a case study analysis, it is too noisy and condensed for this investigation. Therefore, a methodology was derived to reduce the dimensionality of the data so that it represented the key trends for the footfall descriptors.

*K*-means clustering is an unsupervised algorithm that groups unlabelled data into similar clusters based on their features. It was chosen for this study as it summarises the data so that the main variations in footfall descriptors are still maintained yet reduces the dimensionality so that it is more manageable for comparison with footfall data. Other potential methods, such as creating an aggregate measure, could result in the loss of information from the different footfall descriptors which could be key for explaining a footfall trend. In addition, *K-*means clustering is a commonly used and understood methodology in many fields including geodemographic analysis (Burns et al., 2018; Spielman & Singleton, 2015).

The algorithm attempts to minimize the sum of squared Euclidean distance between randomly generated cluster centres and nearby data points (Lloyd, 1982). When the sum of squared distance cannot be minimized and the cluster centres are stationary, the algorithm has converged on a solution. The best solution for a *k*-means clustering is one which generates well-separated and compact clusters which are interpretable within the context of the data.

In order to run the *k*-means algorithm, the features were standardised according to their mean and standard deviation. As *k*-means optimises the sum of squared distance, outliers can have a large impact on the results. Some locations were classed as outliers because they had unusually large or small values for some variables. For example, three sensors in Lymington were removed as they were over 18 km from the nearest entertainment activity. A further five sensors were removed iteratively throughout the clustering process, as they were the furthest point from any cluster centre. The resulting clusters were as compact and well-separated as possible without removing more outliers than necessary.

The features were then checked against each other to ensure there are no high correlations to avoid multicollinearity (see Appendix B).

**Fig. 2:**
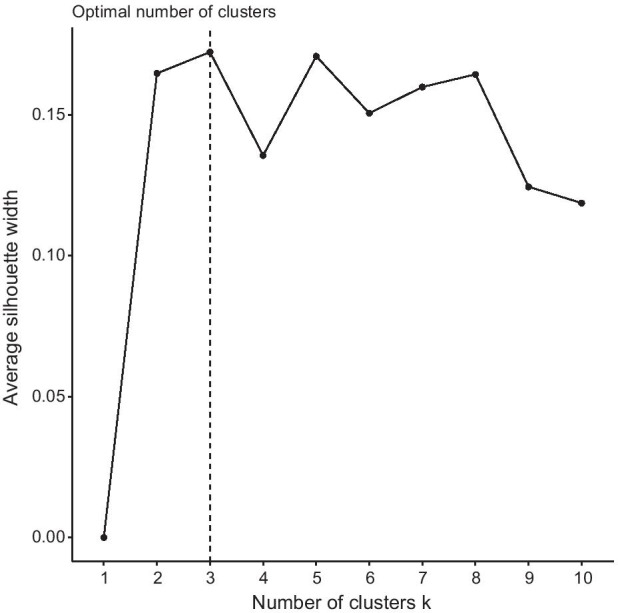
The change in average silhouette score for different values of *k* in *k*-means clustering algorithm

The clustering algorithm was run using *k* = 3. There was no prior indication from the data to suggest a value of *k* therefore a comparison of average silhouette score was used. A silhouette score is a measure of how well a certain point fits within the cluster it has been assigned. It ranges from + 1 which represents a point which fits perfectly in the generated cluster, to -1 which represents a point which poorly fits into the current cluster and would fit better in another. The average silhouette score is defined as the mean silhouette score for every point in the clustering. The average silhouette score for different values of *k*, as shown in Fig. 2, were used to determine that *k* = 3 provides the best separation and cluster results.

One of the pitfalls of using this method is that it is a stochastic process. Therefore, if certain cluster centres were generated in an unfavourable position then it could lead to a poor result. To avoid this issue, the clustering was optimised using 10,000 runs with different randomly generated starting centres to find the best clustering outcome.

The average silhouette score for the final clustering was 0.17. Although this is quite low, this is a result of the ambiguous nature of boundaries between retail areas. It is rare to find a street or micro-location which only serves one purpose and there is often qualities or retailers in a location which cater to a different function than others. In addition, even if there are streets which serve similar purposes, it is unlikely that they will also have the same structural qualities. Therefore, it is understandable that the clusters have a degree of overlap between them. There are methods which tailor to this quality in datasets, notably fuzzy c-means clustering, however they do not produce the clear-cut labels which will be useful when comparing the clusters to their average footfall signature.

## Results

### Cluster Derivations

Cluster profiles often referred to as ‘Pen Portraits’ were then obtained based on values of the cluster centres and exploratory research into individual locations (see Appendix D). The values for the cluster centres and the within sum of squares can be found in Appendix D. The three clusters derived in our analysis were titled *chain and comparison retail micro-locations, business and independent micro-locations* and *value-orientated convenience retail micro-locations*.

**Chain and Comparison Retail Micro-locations [CCR]**

*Number of Sensors: 343 (54%)*

The CCR cluster was the most common of the three clusters and almost every city or town in the sample had a sensor in this cluster. They are named after their predominantly comparison retail function and their dominance towards chain retailers. From the clustering features, these micro-locations had a low proportion of independent retailers, were close to anchor stores and premium retailers and had a bias towards retail outlets over services. As such, destination shopping locations fit well into this cluster, for example, Oxford Street in London, Liverpool ONE in Liverpool and Queen Street in Cardiff. These locations are designed for comparison goods shopping, with a range of chain stores catering to create a large retail offer. These are sought after locations for retailers, often in the retail core of major cities.

**Business and Independent Micro-locations [BI]**

*Number of sensors: 254 (40%)*

The BI cluster encompasses places with a tendency towards independent retail, often in financial and office-dominated districts. 212 (83%) of the sensors in this cluster are sensors in London, representing 70% of the total sensors in London. This cluster captures the employment areas and the destination for many commuters. These areas are common in larger cities, where people do not tend to live near where they work, explaining why this cluster is predominant in London. In terms of the clustering features, BI micro-locations have a high working population, are close to transport hubs and have a high proportion of independent retailers. Some examples of these places are Holborn and the City of London, in London and NOMA and Spinningfields in Manchester. This cluster also includes places which also have a high proportion of night-time economy outlets such as Park Street in Bristol, Soho in London and Bold Street in Liverpool.

A significant distinction of locations in this cluster is that they have 9% more restaurants than the average British high street, subsequently reflected in a near 1:1 ratio between service and retail outlets. This shows that this cluster has a more experience-based function than a comparison retail-based one. This is supported by their large distance from anchor stores, and their small proportion of value retailers.

**Value-orientated Convenience Retail Micro-locations [VOCR]**

*Number of Sensors: 43 (7%)*

The VOCR micro-locations cluster describes smaller, secondary centres of a larger urban area. These are more residential areas with a high prevalence of budget convenience retailers and betting and charity shops. They are defined by their higher proportion of value outlets, their larger distance from premium stores and entertainment venues and their low workplace population. These areas are the opposite of destination shopping areas; people visit these areas out of convenience. They exist due to their accessible location near to residential areas so that consumers can gather their essentials without making a longer trip. VOCR micro-locations have few entertainment venues and night-time economy outlets, as these are things which people are willing to travel for. Some examples of locations which fit into this cluster are Penge, Wood Green and Kilburn in London, Orpington, Shirley in Southampton, and Blatchington Road in Brighton. VOCR micro-locations also have the most vacant units, suggesting that they struggle to find retailers to fill stores. Another feature of this cluster is a distinctly higher proportion of charity shops. 5.9% of the nearest 25 stores to each sensor in this cluster were charity shops, compared to 1.8% in the CCR cluster and 0.6% in the BI cluster and 4.3% greater than the average for England and Wales of 1.6%.

### Cluster Footfall Signature and Magnitude

Footfall measurements are often used as a proxy for retail centre vitality (Coca-Stefaniak, 2013; Millington et al., 2018), however there is limited research quantifying how functional and morphological factors impact footfall magnitude and signature. By investigating the footfall patterns exhibited by these clusters built on functional and morphological characteristics, a greater understanding of variations in footfall magnitude and signature can be achieved.

**Fig. 3:**
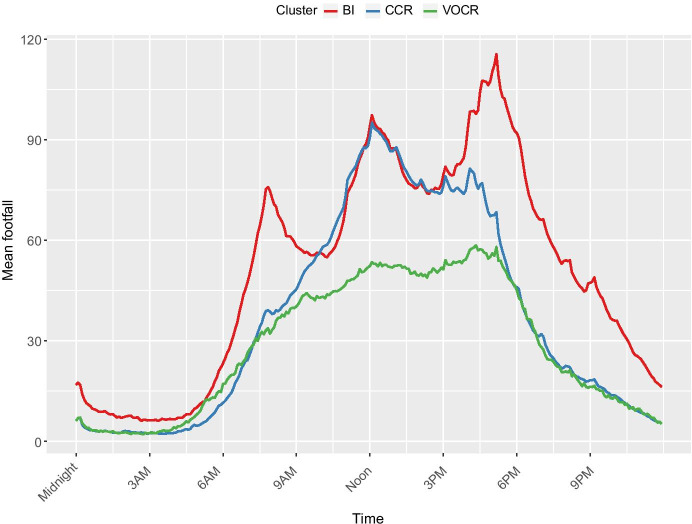
Average footfall distribution for each cluster for a weekday (Monday to Friday) to 5-min accuracy

**Fig. 4:**
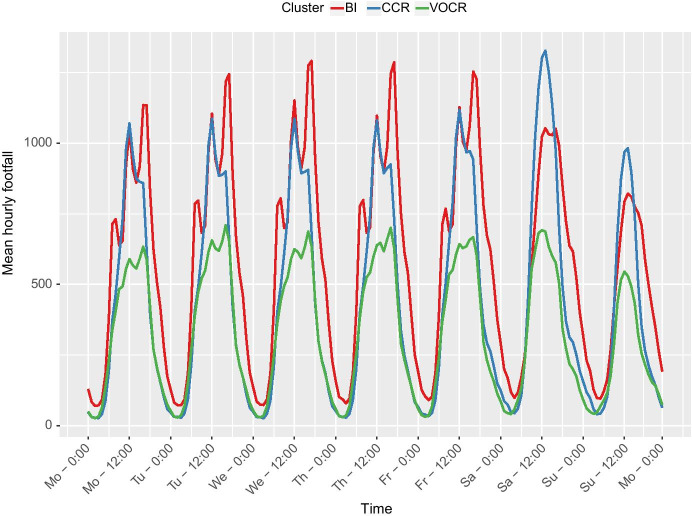
Average footfall across a week for each cluster to hourly accuracy

Footfall measurements from January 2017 until August 2018 were averaged across the locations in each cluster to investigate whether the different functions and characteristics of the micro-location impact footfall. Only the sensors with footfall data for 75% of a full year were used to remove any bias from new or temporary sensors which only have footfall data for potentially busier or quieter times of the year. This removed 12 sensors from the sample. Descriptive statistics were calculated for the average week (by hour), and average weekday (by 5 min) for each cluster as shown in Table 2 and Figs. 3 and 4.

**Table 2:** Summary statistics for footfall (people per 5 min) across the clusters

Statistic		CCR micro-locations	BI micro-locations	VOCR micro-locations
Maximum:	Mon	94 @ 12:05	106 @ 17:10	55 @ 16:15
	Tues	95 @ 12:05	117 @ 17:10	61 @ 16:20
	Wed	96 @ 12:05	121 @ 17:10	61 @ 17:10
	Thurs	95 @ 12:05	119 @ 17:10	62 @ 16:20
	Fri	98 @ 12:05	113 @ 17:10	57 @ 16:20
	Sat	116 @ 13:05	92 @ 13:05	60 @ 13:25
	Sun	86 @ 13:05	71 @ 14:05	47 @12:05
Weekly Mean		37	49	27
Standard Deviation		32	31	19

Figure 3 shows that early in the morning on weekdays, the BI micro-locations have higher footfall than CCR micro-locations. Although by 10:00, the CCR micro-locations are just as busy, and both rise in footfall until 12:05. This maximum weekday peak is consistent at 94–101 people per 5 min for CCR and BI micro-locations. Footfall in CCR micro-locations then decreases into the afternoon and evening, whereas footfall in BI micro-locations experiences a 14:00 lull before peaking again into the early evening. This is reflected through the consistent 17:10 maximum footfall values for BI micro-locations of 106–121 people per 5 min, shown in Table 2. During the evening, this cluster is the busiest, keeping over 25 people per 5 min until past 22:00 and never dropping below 5 people per 5 min. BI micro-locations have a distinctive weekday footfall pattern consisting of three peaks at 8:00, 12:00 and 17:00.

The VOCR micro-locations have the lowest average footfall of all the clusters, and they are never the busiest. Their maximum value is 62 people per 5 min, which is just over half the size of the maximum values for the other clusters. The footfall signature of VOCR micro-locations is hump shaped, slowly increasing from 5:00 to 16:15 – 17:10, where it peaks on weekdays. After then, footfall decreases exponentially to under 10 people per 5 min by 22:30.

As visible in Fig. 4, CCR micro-locations are significantly busier on Saturdays compared to the weekdays, with their maximum footfall of 116 people per 5 min at 13:05 that day. Although CCR micro-locations have the highest peak, BI micro-locations have the highest consistency, with a mean footfall of 49 people per 5 min, compared to 37 people per 5 min. However, VOCR micro-locations have the lowest standard deviation, showing that, although their average footfall is low, it is the most consistent throughout the day and throughout the week.

VOCR micro-locations have very similar footfall signatures during the weekend as the weekday, in contrast BI micro-locations have very different footfall signatures. They have lower footfall at weekends, peaking at 92 people per 5 min at 13:05 and do not exhibit the three peak structure previously observed, instead showing a peak at early afternoon with a slow drop into evening when they the only cluster to retain significant footfall into the night. Friday and Saturday nights appear to be the busiest nights, staying at above 25 people per 5 min until after 00:00. In contrast, the other clusters have dropped below this threshold by 21:00. Sunday is the quietest day for every cluster even the most consistent VOCR micro-locations, exhibit a smaller peak on this day.

## Discussion

This study has produced three distinct clusters of retail micro-locations which vary in terms of their function and morphology: chain and comparison retail micro-locations [CCR], business and independent micro-locations [BI], and value-orientated convenience retail micro-locations [VOCR]. When the average weekly and daily footfall patterns of these clusters were investigated, distinct patterns in signature and magnitude were evident. These differences in footfall signature and magnitude can be partially explained by various characteristics of the retail micro-location, essentially their form and function.

Firstly, the CCR micro-locations exhibited a footfall pattern with the busiest times on Saturdays, and during daytime hours from late morning to early afternoon. This reflects this cluster’s prominent comparison retail function indicated by its low service to retail ratio and the low proportion of independent stores in the clustering. For the majority of people, Saturday is a day of leisure when they have ample free time. Comparison retail tends to be recreational and time consumptive (Guy, 1998), therefore supporting the link between this function and significant Saturday and daytime footfall. In addition, this cluster has the highest average density of retail units showing that the retail offer is more condensed in these micro-locations, therefore, increasing the overall footfall magnitude. Besides, a condensed retail offer has the capacity to encourage linked trips, where consumers visit different locations in the same trip (Wrigley et al., 2009).

In comparison, the BI micro-locations have weekday dominant footfall with three peaks at 8:00, 12:00 and 17:00. This footfall pattern reflects commuting into and out of work, with an additional increase in footfall during a lunch time break, is similar to that observed in other studies (Berry et al., 2016; Lugomer & Longley, 2018). This is further supported by the large workplace population of the cluster and close proximity to transport hubs with many of the sensors located in central London—a destination for many public transport commuters (Lyons & Chatterjee, 2008). The absence of this pattern during the weekend confirms this interpretation and shows the extent to which working population determines footfall in these locations. Furthermore, BI micro-locations retain footfall later into the evening than the other clusters. With a higher than average number of restaurants and bars, these micro locations could be also viewed as attractive leisure and night-time economy destinations (Ravenscroft et al., 2000). However, the amount of footfall in the late evening is significantly less than during the day, demonstrating that, on average, this night-time economy function is supplementary to the workplace function.

The VOCR micro-locations are the quietest and steadiest in terms of footfall. This constant and consistent flow of people could be explained by their convenience-based function as convenience retail is characterised by short and frequent trips (Guy, 1998). The VOCR micro-locations tend to be in residential areas which serve a local demand with a smaller catchment size, therefore generating less footfall. The smaller magnitude of footfall of these micro-locations could be also associated with larger distance to many footfall attractors such as anchor stores, transport hubs and entertainment activities.

However, not all of these footfall patterns can be explained by features of the micro-location. For example, in every cluster Sundays saw 26–32% less footfall compared to the other days of the week, which can be explained by the reduced to 6 h opening hours on this day for stores larger than 280 square metres, imposed by the 1994 Sunday Trading Act (Gov. UK, 2019). Research shows that these large stores can be key footfall attractors and having these stores reduce their opening hours may deter people from visiting their high street on Sundays (Williamson et al., 2006).

These results help to build a clear understanding of how and why footfall fluctuates throughout the day and week and better understand its relationship with micro-location characteristics. In general, these results show that footfall and, as an extension of that, retail vitality, vary temporally and spatially on a micro-locational scale as a result of multiple external and internal influences. More specifically, this study shows some key drivers of footfall at a micro-location level: anchor stores, workplace population, density of retail units and distance to transport hubs. However, it would be incorrect to assume that all retailers within a particular retail centre benefit equally from the increased footfall in terms of spend, as that depends on many other factors on a micro-location level (Millington et al., 2015). This supports strategies to increase high street vitality which are holistic and consider this complexity of micro-locational factors within the wider retail centre. Footfall is often used as an indicator of high street vitality therefore a better understanding of it, underpinned by reliable data and robust empirical analysis is vital for business, academia and policy makers.

### Implications

The results of this study pertaining to variation in footfall magnitude, signature and the function and form of the particular retail micro-location have a number of implications for various stakeholders. Firstly, it supports revitalisation and town centre strategies which consider the complexity of micro-locational influences within a retail centre, as this study has shown the importance of these factors in determining footfall and retail centre vitality. This is particularly relevant as footfall is widely used as a measure for retail centre performance, therefore having a clearer understanding of how and why it fluctuates would be beneficial. Understanding these factors can be valuable for retailers and planners in managing pedestrian flows, setting effective opening hours and investing in ideas which would be attractive to their target consumer. For example, BI micro-locations have a significantly bigger daytime footfall than evening footfall, despite its night-time economy. This knowledge could be used to develop schemes to increase the dwell time of daytime population and encourage them to support the night-time economy establishments, increasing the retail resilience of the area.

Secondly, these results have demonstrated the potential of using morphological and functional characteristics to predict footfall for areas where there are not sensors. Although these clusters are generalisations of micro-locations, they begin to draw out patterns between certain characteristics and spatial and temporal footfall variations. With technological advancements increasing the wealth of data on urban characteristics and mobilities and the development of algorithms capable of processing this data, there is potential for these kinds of patterns to be used to predict footfall for all retail areas. This would be a useful tool for benchmarking and location planning, managing pedestrian flows and business logistics such as opening hours and staffing.

Thirdly, this study has contributed to a more comprehensive understanding of retail mobilities. Although many footfall determinants have been identified in literature, how they impact footfall temporally is not always investigated or quantitatively shown. This paper has demonstrated how different micro-locational characteristics impact footfall to 5-min intervals throughout an average week which provides new insight into footfall determinants and urban mobility as a whole.

### Limitations

There are some limitations which have to be considered when examining and applying the results of this study, in addition to the data limitations discussed previously. Firstly, the sample size of 640 micro-locations for Great Britain is relatively small, with a bias towards London and the south of England. 52% of sensors are in the Greater London region, which has been shown to exhibit unique footfall patterns when compared to the nation as a whole (Mumford et al., 2017). Further, there are disproportionally fewer sensors in mid-sized centres and smaller centres, particularly in the north of England and Wales. Mid-sized retail centres and northern retail centres have been identified as the worst affected by unfavourable changes in the retail sector (Millington et al., 2015; Wrigley & Dolega, 2011). In addition, the sensors are predominantly located in city centre environments, as opposed to suburban high streets or district centres, which face their own unique challenges to their future retail vitality and viability (Griffiths et al., 2008). As such the data sample is skewed towards micro-locations in larger urban areas that tend to be more successful and sustainable retail destinations, potentially with lower vacancy rates and steady footfall.

Secondly, although this study has grouped each of the micro-locations into three clusters, they may not be as clearly delineated in reality. Cluster analysis is a well-established and widely used form of analysis, however its outputs are a representation determined by decisions made by the researcher, which, if made differently would produce alternate and yet still valid results (Vickers & Rees, 2007). This inherent quality of clustering techniques means that these micro-locations are more complex than the cluster descriptions. This is evident through the variation of footfall signatures within each cluster. The distributions shown in Figs. 3 and 4 are the averages for all the sensors within that cluster and they may not reflect all micro-locations in that cluster. Some of the sensors may have somewhat different footfall magnitudes and signatures compared to the average in their cluster, despite overall similarity of a particular cluster functional and morphological characteristics.

Finally, due to the aforementioned bias in the availability of footfall data, it is likely that there are other identifiable micro-locations clusters in the wider country which have not been represented by this study. For instance, in Mumford et al. (2020) four types of town were identified based on their monthly footfall patterns: comparison, holiday, speciality and multi-functional. It is apparent that our sample is biased towards Mumford et al.’s comparison centres overlooking the different micro-locational patterns that could exist in the remaining clusters, such as seasonal popularity, tourism and non-retail anchors (Mumford et al., 2020; Newing et al., 2018).

## Conclusion

In conclusion, this study has provided a novel application of sensor data to better understand retail behaviours and footfall. It has shown that patterns in the magnitude and signature of footfall data, and by extension retail vitality, can be to an extent, explained by functional and morphological characteristics of the micro-location. In particular, the ability of key footfall attractors such as anchor stores and transport hubs to significantly drive footfall at certain times throughout the day and week. This paper has also demonstrated the importance of the type of retail offer, comparison, convenience or recreational, on the magnitude and signature of footfall within the micro-location. The results display three clear narratives of micro-location morphology, function and footfall distribution, which aid greater understanding of the interrelationship and patterns that exist between them. Although the value added by this study is clear, it needs to be highlighted that the identified clusters are merely a representation of the more complex real world and any application of these narratives to a unique micro-location should consider the different functions which that place represents (Millington et al., 2015).

Finally, future research will benefit from employing more footfall data to facilitate investigation into monthly, annual and longer-term trends in footfall and how those could relate to functional and morphological characteristics. In this study we present the potential for functional and morphological characteristics of micro-locations as a predictor for footfall in locations where footfall is not measured. Being able to model footfall for an entire retail centre could be invaluable for decision-making, urban planning and for retail location planning.

## Data Availability

Available on request*

## Appendix A

Distribution of sensor sample across UK towns and cities.

**Table:**

Town/City	Number of sensors (n = 640)	% of sample
Birmingham	5	0.8%
Blackpool	5	0.8%
Boston	2	0.3%
Bradford	2	0.3%
Brighton	19	3.0%
Bristol	14	2.2%
Bromley	11	1.7%
Cambridge	11	1.7%
Cardiff	8	1.3%
Chelmsford	3	0.5%
Chester	18	2.8%
Croydon	6	0.9%
Dorchester	7	1.1%
Gateshead	1	0.2%
Gloucester	14	2.2%
Hove	3	0.5%
Hull	5	0.8%
Kingston Upon Thames	20	3.1%
Leamington Spa	8	1.3%
Leeds	13	2.0%
Leicester	6	0.9%
Liverpool	16	2.5%
London	291	45.5%
Manchester	18	2.8%
Market Harborough	13	2.0%
Newcastle Upon Tyne	7	1.1%
Norwich	14	2.2%
Nottingham	17	2.7%
Orpington	6	0.9%
Oxford	11	1.7%
Plymouth	8	1.3%
Reading	17	2.7%
Salisbury	11	1.7%
Sheffield	8	1.3%
Solihull	2	0.3%
Southampton	8	1.3%
Taunton	6	0.9%
Watford	3	0.5%
Windsor	1	0.2%
York	2	0.3%

## Appendix B

Correlation coefficients and significance of the micro-locational footfall influencers used in the clustering.

**Table:**

	Premium Stores	Entertainment Venues	Anchor Stores	Workplace Population	Transport Hubs	Car Parks	Density of units	Value Stores	Independent Stores	Night-time Economy	Service prominence	Vacancy Rate	Centrality
Premium Stores	1.00												
Entertainment Venues	0.25 ^a^	1.00											
Anchor Stores	0.24 ^a^	0.41 ^a^	1.00										
Workplace Population	-0.26 ^a^	-0.06 ^b^	-0.07 ^c^	1.00									
Transport Hubs	0.19 ^a^	0.26 ^a^	-0.02 ^c^	-0.19 ^a^	1.00								
Car Parks	0.07 ^c^	0.09 ^b^	0.19 ^a^	0.11 ^a^	-0.12 ^a^	1.00							
Density of units	-0.23 ^a^	-0.20 ^a^	-0.28 ^a^	-0.06 ^b^	0.06 ^b^	-0.14 ^a^	1.00						
Value Stores	0.43 ^a^	0.03 ^b^	-0.04 ^c^	-0.33 ^a^	0.18 ^a^	0.02 ^c^	-0.08 ^b^	1.00					
Independent Stores	0.24 ^a^	0.11 ^a^	0.44 ^a^	-0.18 ^a^	-0.03 ^c^	0.07 ^c^	-0.16 ^a^	0.05 ^c^	1.00				
Night-time Economy	-0.07 ^c^	-0.09 ^b^	0.29 ^a^	0.34 ^a^	-0.18 ^a^	0.09 ^c^	-0.33 ^a^	-0.27 ^a^	0.27 ^a^	1.00			
Service prominence	0.09 ^b^	0.11 ^a^	0.20 ^a^	-0.05 ^b^	-0.01 ^c^	0.08 ^b^	-0.30 ^a^	0.01 ^c^	0.27 ^a^	0.25 ^a^	1.00		
Vacancy Rate	-0.10 ^b^	-0.04 ^b^	-0.09 ^b^	-0.09 ^b^	0.13 ^a^	-0.10 ^b^	0.09 ^b^	0.11 ^a^	-0.09 ^b^	-0.18 ^a^	0.00 ^c^	1.00	
Centrality	0.40 ^a^	0.32 ^a^	0.14 ^a^	-0.41 ^a^	0.29 ^a^	-0.03 ^c^	0.02 ^c^	0.30 ^a^	0.00 ^c^	-0.30 ^a^	0.26 ^a^	0.05 ^c^	1.00
^a^ correlation significant to 0.01; ^b^ correlation significant to 0.05; ^c^ correlation not significant													

## Appendix C

Specifics for the derivation of some of the footfall descriptors, informed by the conditions in Dolega et al. (2016)

### Value Stores

Store Name: Aldi, Lidl, Iceland, Primark, Farmfoods, Poundworld, Poundstretcher, Home Bargains, Savers, B&M Bargains, Pound Bakery

Category: Discount & Surplus Stores, Charity And Secondhand Shops, Pawnbroking And Cheque Cashing

Subcategory: Bookmakers.

### Night-time Economy Locations

Category: Bars Pubs And Clubs, Off Licenses And Restaurants.

Subcategory: Fast Food Takeaway, Take Away Food Shops, Fish And Chip Shops, Pizza Takeaway, Chinese Fast Food Takeaway, Indian Takeaway, Fast Food Delivery, Amusement Parks & Arcades, Theatres & Concert Halls, Cinemas, Snooker, Billiards & Pool Halls, Bowling Alleys

### Ratio of Service to Retail

Retail over service where retail is:

Classification: Comparison

Category: Groceries, Supermarkets & Food Shops, Bakers, Confectionery, Tobacco, Newsagents, Off Licenses, Butchers & Fishmongers

And service is:

Classification: Service.

### Anchor Stores

Store name: Tesco (excluding Express and in store services), Sainsburys (excluding Local and in store services), Waitrose (excluding Little Waitrose), Morrisons (excluding in store services), ASDA (excluding in store services), John Lewis, Debenhams, Marks & Spencer, Harvey Nichols, H&M, Primark, Zara, Boots, Next, B&Q and House of Fraser.

### Premium Stores

Store name: Waitrose, John Lewis, Harvey Nichols, Laura Ashley, Ted Baker, Tommy Hilfiger, Fat Face, Superdry, Seasalt, Jack Wills, White Stuff, Crew Clothing, Boss, Cath Kidston, Joules, Swarovski, Lacoste, Diesel, Apple Store, Bose, Hotel Chocolat, Radley, Karen Millan, Michael Kors, The White Company, Reiss, All Saints, Tessuti, Flannels, Ralph Lauren, Kate Spade, Mulberry, Burberry, Armani, Calvin Klein, Coach, Dune, Diesel, Fossil, Fred Perry, French Connection, Guess, Hobbs, Karl Lagerfeld, Kurt Geiger, L'Occitane, Lacoste, Levi, Lindt, Osprey, Swarovski, Timberland and Toms

### Entertainment Activities

Subcategory: Cinemas, Theatres & Concert Halls, Amusement Parks & Arcades, Museums & Art Galleries, Sports Grounds & Stadiums, Tourist Attractions, Party Venues & Function Rooms, Bingo Halls, Bowling Alleys, Ticket Outlets & Box Offices, Golf Courses, Snooker, Billiards & Pool Halls, Driving Ranges, Ice Rinks, Booking Agents, Paintball & Combat Games and Karting

## Appendix D

Values for final cluster centroids, un-standardised for comprehensibility.

**Table:**

	CCR micro-locations	BI micro-locations	VOCR micro-locations
Within cluster sum of squares	2130	2340	1951
Number of observations	343	254	43
Distance to nearest anchor store (m)	85.98	199.93	216.53
Distance to nearest premium store (m)	122.79	234.07	1860.41
Distance to nearest entertainment activity (m)	120.79	157.09	328.91
Mean workplace population	409.03	770.28	94.07
Distance to nearest transport hub (m)	159.36	93.79	249.72
Distance to nearest car park (m)	160.69	227.58	276.44
Density of units (unit per 100^2^π m^2^)	79.38	48.86	52.63
Proportion of value stores	5%	2%	12%
Proportion of independent stores	38%	55%	58%
Proportion of night-time economy stores	15%	34%	15%
Ratio of service to retail	0.45	0.87	0.84
Proportion of vacant stores	9%	5%	6%
Centrality of street	0.08	0.05	0.16

The prevalence of each category of store as defined by LDC’s survey (2017) for each cluster and for the entire sample (*n* = 222,953). The nearest 25 stores to each sensor were considered when calculating a total percentage for the cluster. This information was used alongside the cluster centroids in Appendix C to create the cluster pen portraits.

**Table:**

LDC Categories	CCR micro-locations (%)	BI micro-locations (%)	VOCR micro-locations (%)	Entire sample (%)
Accommodation	0.5	3.3	0.0	1.9
Auto & Accessories	0.0	0.0	0.2	0.3
Auto Services	0.0	0.1	0.5	1.5
Bakers	1.2	1.1	1.0	1.0
Banks, Financial Services & Building Societies	4.2	3.0	3.4	1.7
Bars, Pubs & Clubs	3.0	7.8	2.1	4.6
Books, Arts & Crafts, Stationery, Printers	2.8	3.0	2.3	1.9
Butchers & Fishmongers	0.3	0.1	2.4	0.6
Cafes & Fast Food	8.6	15.2	9.7	10.7
Car & Motorbike Showrooms	0.0	0.0	0.0	0.7
Charity & Secondhand Shops	1.8	0.6	5.9	1.6
Chemists, Toiletries & Health	4.1	2.0	3.2	2.6
Confectionery, Tobacco, Newsagents	2.3	1.8	0.9	1.9
Department Stores & Mail Order	0.8	0.3	0.0	0.3
Discount & Surplus Stores	0.5	0.0	2.6	0.6
DIY, Hardware, Builder's Merchants & Household Goods	0.7	0.6	0.7	1.7
Electrical Goods & Home Entertainment	5.4	2.7	5.0	2.6
Employment & Post Offices	1.4	0.8	1.5	1.5
Entertainment	2.5	3.3	4.0	2.5
Estate Agents & Auctioneers	0.9	2.1	3.1	2.9
Fashion & General Clothing	15.1	6.9	5.3	4.6
Florists & Garden	0.2	0.3	0.2	0.5
Footwear	3.0	0.7	1.2	0.7
Furniture, Carpets, Textiles, Bathrooms & Kitchens	1.8	1.2	2.4	3.0
Gifts, China & Leather Goods	1.4	1.5	0.6	0.8
Groceries, Supermarkets & Food Shops	2.5	3.7	6.8	6.9
Hairdressing, Health & Beauty	8.3	7.7	13.8	10.8
Household & Home	0.1	0.1	0.0	0.6
Jewellers, Clocks & Watches	3.1	0.9	1.0	1.1
Launderettes, Dry Cleaners & Other	0.4	1.0	0.8	1.1
Locksmiths, Clothing Alterations & Shoe Repairs	0.6	0.5	0.6	0.4
Medical	0.3	0.4	0.0	0.8
Miscellaneous	0.6	0.8	1.4	1.6
Non-Retail	1.5	2.3	3.3	3.5
Off Licences	0.2	0.6	0.8	0.6
Pawnbroking & Cheque Cashing	0.4	0.2	1.3	0.4
Pet Shops & Pet Supplies	0.0	0.0	0.0	0.3
Petrol Filling Stations	0.0	0.0	0.0	0.7
Restaurants	4.7	14.8	3.7	5.8
Royal Mail Delivery Offices	0.0	0.0	0.0	0.1
Shopping Centres & Markets	0.3	0.1	0.4	0.1
Sports, Toys, Cycle Shops & Hobbies	3.6	1.3	1.3	1.4
Transport	0.5	1.3	0.8	2.3
Travel Agents & Tour Operators	1.5	0.6	0.6	0.6
Vacant	8.9	5.1	5.3	8.3

## Footnotes

1 From a methodological standpoint, a walking network distance would be more appropriate for this analysis than a Euclidean distance. However, due to the COVID-19 pandemic limited access to on-campus resources, the computing capabilities needed to use this measure were unavailable. Preliminary data exploration and the relatively short distances would indicate that using network distance would have negligible overall impact.
